# 

**Published:** 1906-01-15

**Authors:** 


					﻿Vol. LX.	JANUARY 15, 1906.	No. 1.
THE
DENTAL REGISTER
EDITED BY
NELVILLE S. HOFF, D.D.S/, \
ANN ARBOR, MICH.	I |
: PRICE, ONE DOLLAR A YEAR IN ADVANCF.
Published Monthly by
SAMUEL A. CROCKER & CO.,
Ohio Oental and Surgical Depot,
35 to 39 W. 5th St., Cincinnati, 0.
Entered at the Pnstoffice at Cincinnati, O , as second-class mail matter.
				

